# Circulation of SARS-CoV-2 Variants among Children from November 2020 to January 2022 in Trieste (Italy)

**DOI:** 10.3390/microorganisms10030612

**Published:** 2022-03-14

**Authors:** Maria Dolci, Lucia Signorini, Carolina Cason, Giuseppina Campisciano, Paolo Kunderfranco, Elena Pariani, Cristina Galli, Vincenzo Petix, Pasquale Ferrante, Serena Delbue, Manola Comar

**Affiliations:** 1 Department of Biomedical, Surgical and Dental Sciences, University of Milan, Via Pascal 36, 20133 Milan, Italy; maria.dolci@unimi.it (M.D.); lucia.signorini@unimi.it (L.S.); 2 SSD of Advanced Microbiology Diagnosis and Translational Research, Institute for Maternal and Child Health-IRCCS Burlo Garofolo, Via dell’Istria 65/1, 34137 Trieste, Italy; carolina.cason@burlo.trieste.it (C.C.); giusi.campisciano@burlo.trieste.it (G.C.); vincenzo.petix@burlo.trieste.it (V.P.); manola.comar@burlo.trieste.it (M.C.); 3 Bioinformatics Unit, Humanitas Clinical and Research Center—IRCCS, Via Alessandro Manzoni 56, 20089 Milan, Italy; paolo.kunderfranco@humanitasresearch.it; 4 Department of Biomedical Sciences for Health, University of Milano, Via Pascal 36, 20133 Milan, Italy; elena.pariani@unimi.it (E.P.); cristina.galli@unimi.it (C.G.); 5 Istituto Clinico Città Studi, Via Niccolò Jommelli, 17, 20131 Milan, Italy; pasquale.ferrante@unimi.it; 6 Department of Medical Sciences (DSM), University of Trieste, 34129 Trieste, Italy

**Keywords:** pediatric population, SARS-CoV-2 infection, variant circulation

## Abstract

Introduction: The ongoing coronavirus disease 19 (COVID-19) outbreak involves the pediatric population, but to date, few reports have investigated the circulation of variants among children. Material and Methods: In this retrospective study, non-hospitalized pediatric patients with SARS-CoV-2-positive nasopharyngeal swabs (NPS) were enrolled at the Institute for Maternal and Child Health-IRCCS Burlo Garofolo, Trieste (Italy), from November 2020 to January 2022. SARS-CoV-2 variants were identified by in vitro viral isolation, amplification, automatic sequencing of the receptor binding domain (RBD) of the SARS-CoV-2 spike coding gene, and subsequent next-generation sequencing. The growth curves of the isolated strains were defined in vitro by infecting Vero-E6 cells and quantifying the viral load in the supernatants up to 72 h post-infection by qRT–PCR. The neutralization activity of sera obtained from a COVID-19 vaccinated subject, recovered (2020) patient, vaccinated and recovered (2021) patient, and seronegative subject was assessed by microneutralization assay against the different variants. Results: In total, 32 SARS-CoV-2-positive children, 16 (50%) females, with a median age of 1.4 years (range: 1 day–13 years), were enrolled. The D614G amino acid substitution was detected in all isolated and amplified viral strains. Of the 32 isolates, 4 (12.5%) carried a nonsynonymous nucleotide mutation leading to the N439K (3/4), lineage B.1.258 (∆H69/∆V70), and S477N (1/4) substitution. In 7/32 (21.8%) isolates, amino acid substitutions allowed the identification of a delta variant, lineage B.1.617.2-AY.43, and in 1/32 (3.1%), the Omicron strain (B.1.1.529.BA1) was identified. The growth curves of the B.1, B.1.258 (∆H69/∆V70), B.1.617.2-AY.43, and B.1.1.529.BA1 variants did not show any significant differences. A reduction in the serum neutralizing activity against B.1.258 (∆H69/∆V70) only in a vaccinated subject (1.7-fold difference), against B.1.617.2-AY.43 in a vaccinated subject and in recovered patients (12.7 and ≥2.5-fold differences, respectively), and against B.1.1.529.BA1 variant (57.6- and 1.4-fold differences in vaccinated and in vaccinated and recovered patients) were observed compared to the B.1 variant. Conclusions: SARS-CoV-2 variants carrying the B.1.258 (∆H69/∆V70) and S477N substitutions were reported here in a pediatric population for the first time. Although the growth rates of the isolated strains (B.1.258, B.1.617.2-AY.43, B.1.1.529.BA1) did not differ from the B.1 variant, neutralizing activity of the sera from vaccinated subjects significantly decreased against these variants. Attention should be devoted to the pediatric population to prevent the spread of new SARS-CoV-2 variants in an unvaccinated and predominantly naive population.

## 1. Introduction

The ongoing coronavirus disease 19 (COVID-19) outbreak is caused by the severe acute respiratory syndrome coronavirus-2 (SARS-CoV-2). The first cases of atypical pneumonia were reported on 31 December 2019, in Wuhan, China, and as of 26 December 2021, 281 million cases and 5.41 million deaths have been reported worldwide [1,2]. Regarding the pediatric population, few studies have indicated the infection rate among children, and 1–10% of total COVID-19 cases have been observed in this population [3,4,5]. The low rate of SARS-CoV-2 detection via nasopharyngeal swabs (NPS) may be due to mild/asymptomatic infection that escapes detection, the lower susceptibility to infection among children than adults (as children express fewer ACE2 receptors), or the prioritization of testing adults [6,7,8]. A multinational, multicenter cohort study conducted among 25 different countries in Europe provided an overview of the clinical impact of SARS-CoV-2 infection. The study found that COVID-19 is milder and has lower hospitalization rates among children than adults [9]. Regarding neonates, SARS-CoV-2 infection seems to be mainly transmitted from mothers, even if vertical transmission is still unclear [10,11]. Family clusters might be the main source of infection for children [12], while educational settings increase the circulation of the virus among adolescents [13]. In addition to adults, children are a reservoir for SARS-CoV-2 transmission, even if they are asymptomatic or affected by mild illness, thereby promoting the evolution of the virus [14,15,16]. To date, few studies have examined the circulation of SARS-CoV-2 variants of concern (VOCs) and variants of interest (VOIs) in the pediatric population. In Israel, when the alpha variant circulated from December 2020 to February 2021, the virus transmission rate increased among children under 10 years of age [13]. In contrast, in a study conducted by Howard-Jones, the SARS-CoV-2 Delta strain was shown to have a lower level of transmission among children than among adults [17]. To obtain more data on the circulation of SARS-CoV-2 variants in children, the current retrospective study examined 32 pediatric SARS-CoV-2-positive patients with nasal-pharyngeal swabs (NSPs) collected between 18 November 2020, and 7 January 2022, at the Institute for Maternal and Child Health-IRCCS Burlo Garofolo, Trieste, Friuli-Venezia Giulia, Italy. The in vitro growth curves of the isolated strains were assessed, and the microneutralization activity of antibodies elicited by the COVID-19 vaccine and natural antibodies after infection against these variants was evaluated.

## 2. Materials and Methods

### 2.1. Study Design

From 18 November 2020 to 7 January 2022, 32 NPSs were positive for SARS-CoV-2 molecular detection and collected from 32 non-hospitalized children at the Institute for Maternal and Child Health-IRCCS Burlo Garofolo, Trieste, Friuli-Venezia Giulia, Italy. Ethical approval was obtained from the local committee (IRB-Burlo Garofolo IRCCS Trieste, Approval Code: 18.11.20, Approval Date: September 2020), and informed consent was signed by the patients’ parents.

### 2.2. RNA Isolation from NPS and SARS-CoV-2 Detection

Automated extraction of viral RNA was performed from 200 μL of NPS using the commercial Maxwell^®^ RSC Viral Total Nucleic Acid Purification Kit (Promega, Madison, WI, USA), according to the manufacturer’s instructions. The presence of the SARS-CoV-2 genome was evaluated by real-time PCR using the Neoplex™ COVID-19 Detection Kit (Genematrix, Korea), which simultaneously targeted the RdRp and N viral genes. The test was performed according to the manufacturer’s instructions. The reaction was made up in 20 μL, containing 5 μL of 1-step master mix, 5 μL of COVID-19 PPM, 5 μL of RNase-free water, and 5 μL of RNA. The thermal cycles were as follows: 30 min at 50 °C, 15 min at 95 °C, and 45 cycles at 95 °C for 15 s and at 61 °C for 60 s.

### 2.3. Quantitative Reverse Transcription Real-Time PCR (RT–qPCR)

The SARS-CoV-2 N1 gene amount was assessed by means of a quantitative reverse transcription real-time PCR (qRT–PCR) assay using the Applied Biosystem 7500 Real-Time PCR system instrument (ThermoFisher, Waltham, MA, USA) and the CDC and WHO 2019-Novel Coronavirus (2019-nCoV) Real-Time RT–PCR Diagnostic Panel for the detection of nucleic acids from 2019-nCoV (N1 gene) [18,19] (Table 1). The reaction was made up in 25 μL, containing 0.4 μM forward primer, 0.4 μM reverse primer, 0.1 μM probe, 12.5 μL 2x RT–PCR Buffer, 1 μL 25x RT–PCR Enzyme Mix, and 5 μL of RNA using the AgPath-ID One-Step kit (ThermoFisher, Waltham, MA, USA). The thermal cycles were as follows: 30 min at 45 °C, 10 min at 95 °C, and 45 cycles at 95 °C for 15 s and at 55 °C for 30 s. The standard curve was constructed using serial dilution of the pEX-A128-nCoV_all plasmid (Eurofins, Luxemburg) containing part of the SARS-CoV-2 genome. Samples were analyzed in duplicate, and a negative control consisting of a mix supplemented with water was added. The limit of viral genome detection was evaluated as 3 copies/reaction.

### 2.4. Sanger Sequencing of the SARS-CoV-2 Receptor Binding Domain (RBD) Genomic Region

The receptor-binding domain (RBD) of the SARS-CoV-2 spike-encoding region was amplified and sequenced by PCR and subsequent Sanger automatic sequencing. A one-step RT–PCR kit (Thermo Fisher, Waltham, MA, USA) was employed for the amplification step using the set of primers summarized in Table 2. The 50 µL comprised 25 µL of 2× reaction mix, 2 µL of SuperScript III RT/Platinum Taq Mix (Thermo Fisher, Waltham, MA, USA), 1 µL of forward primer (10 µM), 1 µL of reverse primer (10 µM), and 5 µL of RNA sample. The PCR steps were as follows: 1 cycle at 50 °C for 15 min and at 95 °C for 2 min, followed by 40 cycles at 95 °C for 15 s, 54 °C for 30 s, and 68 °C for 40 s, followed by a last step at 68 °C for 5 min. The amplification length product (1000 bp) was checked on 1.5% agarose gels in 0.5× TBE buffer by electrophoresis. PCR products were purified using the QIAquick Gel Extraction Kit (QIAgen, Hilden, Germany) in accordance with the manufacturer’s instructions. Automatic sequencing of the fragments was performed by an external facility (Eurofins Genomics, Munich, Germany) using both forward and reverse primers with the Sanger method. The sequence similarity was searched through the NCBI site (USA) using BLAST [20], comparing the amplified fragments to the reference Wuhan-Hu-1 strain (NC_045512.2). The predicted amino acid sequences were obtained by blastx [20] and compared to the Wuhan-Hu-1 RBD protein sequence with blastp [20].

### 2.5. Next Generation Sequencing

RNA samples that underwent to Next-Generation Sequencing (NGS) were quantitated using a Qubit 3.0 Fluorometer (ThermoFisher Scientific, Waltham, MA, USA) and Qubit RNA HS Assay Kit (Invitrogen Q32855). A RIN for each sample was determined by running 2uL on an Agilent 4200 TapeStation using High-Sensitivity RNA Screen Tapes (Agilent 5067-5579). Then, 5-7 ng of each sample was used to performed libraries following the SMART-Seq Stranded Kit User Manual (Takara Bio 634444). Briefly, cDNAs were first generated from all total RNA fragments after RNA fragmentation. Then, Illumina adapters, indexes, and library purification followed. Final library amplification and purification were performed after enzymatically removing ribosomal fragments originating from rRNA molecules using specific probes. Libraries were quantitated using the Qubit dsDNA HS Assay Kit (Invitrogen, Q32854). The average size of each library was determined by running each sample on Agilent 4200 TapeStation using High-Sensitivity D5000 Screen Tapes (Agilent 5067-5593). Each library was diluted to 4 nM in Resuspension Buffer (Illumina). All libraries were pooled at a concentration of 4 nM before denaturing with 0.2 N NaOH for 5 min at RT. The libraries were neutralized using 200 mM Tris-HCl, pH 7 before being diluted to 20 pM in HT1 buffer (Illumina). Finally, denatured libraries were loaded into an Illumina NextSeq 550 v2 High-Output reagent cartridge at a final concentration of 1.4 pM in HT1 buffer. Denatured PhiX control library was also included at 5% concentration. Dual indexes and paired-end 150 bp sequencing was performed on a High-Output flow cell, yielding about 20M read pairs per sample.

### 2.6. Variation Analysis of SARS-CoV-2 Whole Genome Sequencing

NextSeq 550 bcl files were demultiplexed to fastq files with bcl2fastq (v2.20.0.422). Sequencing reads were then processed with the COVID-19 (variation analysis on WGS PE data) available at Galaxy open-source web-based platform (https://usegalaxy.org/, accessed on 20 February 2022). Briefly, reads were processed for adapter trimming with fastp (Galaxy Version 0.20.1+galaxy0) (PMID: 30423086) and mapped against SARS-Cov-2 complete genome sequence (NC_045512.2) with BWA-MEM (Galaxy Version 0.7.17.1) (PMID: 19451168). The location and tagging of duplicate reads were assessed with Picard tool MarkDuplicates (Galaxy Version 2.18.2.2). Reads were then realigned with and added insert indel qualities (Galaxy Version 2.1.5 + galaxy0). Variants were detected with LoFreq variant-caller (PMID: 23066108) (SNVs and indels) with a minimal coverage of 5 reads, a minimum base-calling quality of 30, and a minimum allele frequency of 0.75 (Galaxy Version 2.1.5 + galaxy0). Finally, variant annotation was performed with SnpEff (PMID: 22728672) (Galaxy Version 4.5covid19). The complete galaxy workflow invocation is available upon request.

### 2.7. Phylogenetic Analysis

The S gene sequences included in this study were aligned using the ClustalW program, implemented in the BioEdit software [21]. Intragroup nucleotide identity was calculated using the Sequence Identity Matrix tool of BioEdit software [21]. The mean intragroup nucleotide identities were expressed as crude percentages with the corresponding minimum and maximum range values.

The phylogenetic analysis was conducted by means of MEGA software, version 6.0 [22]. The best substitution model was selected by analyzing the sequence dataset with the Models Tool available in MEGA software, version 6.0 [22]. The phylogenetic tree was constructed by means of the Neighbor-Joining method and Tamura 3-parameter model, including the S gene sequences of SARS-CoV-2 study strains and reference strains retrieved from the Global Initiative on Sharing All Influenza Data (GISAID) EpiFlu database [23]. The robustness of the phylogenetic tree was estimated by means of a bootstrap analyses with 1000 replicates [24]. 

### 2.8. Cell Culture

Vero E6 (ATCC CRL-1586™) cells were maintained in culture with complete medium composed of Dulbecco’s modified Eagle’s medium (EuroClone, Milan, Italy) supplemented with 10% fetal bovine serum (EuroClone, Milan, Italy), 1X penicillin/streptomycin (EuroClone, Milan, Italy) and 2 mM L-glutamine (EuroClone, Milan, Italy).

### 2.9. Virus Isolation

SARS-CoV-2 isolation and expansion were conducted on the Vero E6 cell line, starting from NPS. Briefly, a total of 4 × 10^5^ cells/well were seeded in a 6-well culture multi-well plate and left to adhere overnight in complete medium at 37 °C with 5% CO_2_ until 80% confluence was reached. Cell infection was obtained by adsorption of 500 μL of NPS for 120 min at 37 °C with 5% CO_2_. After 2 h of adsorption, the inoculum was removed, the cells were washed twice with Dulbecco’s phosphate-buffered saline (DPBS) (EuroClone, Italy), and 2 mL of fresh complete medium was added. Vero E6 cells were maintained for 3 days or more, checking for cytopathic effect (CPE) daily. The procedure was carried out in a biosafety level 3 (BSL3) facility. Infected cell medium was then recovered and stored at −80 °C until use.

### 2.10. Plaque Reduction Assay

The virus titer of supernatant from the infected cells was assessed by means of a plaque reduction assay. Briefly, 4 × 10^5^ cells/well were seeded in a 6-well culture multi-well plate and left overnight in complete medium at 37 °C with 5% CO_2_ until 80% confluence was reached. Then, 500 μL of 10-fold serially diluted supernatants was inoculated onto Vero E6 monolayer cells in duplicate for 2 h. The viral inoculum was removed, and cells were overlaid with complete medium with 3% agarose and maintained for 48 h or 72 h at 37 °C with 5% CO_2_. The cell monolayer was then fixed with paraformaldehyde 3.7% and stained with methylene blue. The titer of the virus was calculated as plaque-forming units (PFU) per milliliter (PFU/mL) and used to define the Multiplicity of Infection (MOI) for the subsequent experiments.

### 2.11. Variant Strain Growth Curves: Kinetics of Infection

A total of 4.5 × 10^4^ Vero E6 cells/well were seeded at 80% confluence in a 48-well culture multi-well plate and left overnight in complete medium at 37 °C with 5% CO_2_. Cell infection was performed by inoculating SARS-CoV-2 isolates at aMOI of 0.01 for 2 h at 37 °C with 5% CO_2_. Growth curves were evaluated at 24, 48, and 72 h post-infection by means of RT–qPCR, as described above. Two replicates for each time points were tested.

### 2.12. Tissue Culture Infectious Dose Assay

The 50% Tissue Culture Infectious Dose (TCID_50_) assay was used to measure the infectivity of the isolated SARS-CoV-2. Briefly, virus serial 1:10 dilutions were performed from 1:10 to 1:10^8^. The prepared dilutions were then transferred on Vero E6 cells, seeded the day before, and incubated for 2 h at 37 °C with 5% CO_2._ Then, complete medium was added, and the plates were kept at 37 °C with 5% CO_2_ for 5–7 days. The CPE end-point method was used for the viral titration, and CPE was counted visually. TCID_50_ values were obtained applying the Reed and Muench formula [25,26].

### 2.13. Microneutralization Assay

For the microneutralization assay, 1.5 × 10^4^ cells/well were seeded in a 96-well culture multi-well plate and left overnight in complete medium at 37 °C with 5% CO_2._ A microneutralization assay was carried out using four different serum samples that were collected: (a) 6 months after the infection from a COVID-19 adult patient infected by the B.1 strain and recovered in May 2020; (b) 2 weeks after the administration of the second dose of Comirnaty (Pfizer-BioNTech) vaccine from a uninfected, vaccinated adult subject; (c) from a recently recovered (December 2021) patient (2 weeks after the infection with B.1.1.529) who became infected after receiving the third dose of the Comirnaty (Pfizer-BioNTech) vaccine; (d) from a SARS-CoV-2-seronegative subject. Serum samples were heat-inactivated at 56 °C for 40 min and serially diluted two-fold in complete medium, from 1:2 to 1:1024, in quintuplicate. In total, 200 TCID_50_ of virus was mixed with serum dilutions and incubated at 37 °C with 5% CO_2_ for 1 h. Then, 30 μL of virus-serum mix was seeded on a Vero E6 cell monolayer, grown on 96-well microplates, and incubated at 37 °C with 5% CO_2_ for 2 h. The complete medium was then added to the cells, which were inspected for CPE with an inverted optical microscope 6 days post-infection. A neutralizing antibody titer that reduces the number of infected wells by 50% (NT_50_) was defined by the means of the Reed and Muench method [26,27].

### 2.14. Statistical Analysis

Pearson correlation was performed to evaluate the difference in the variants’ growth curves. NT_50_ values against the isolated variants were analyzed using the Mann–Whitney nonparametric test. *p* values < 0.05 were considered significant.

The phylogenetic relationship between study strains was assessed by calculating the pairwise *p*-distance implemented in the MEGA software, version 6.0 [22]. The mean nucleotide genetic distances were expressed as crude rates with the corresponding standard deviation (sd).

## 3. Results

### 3.1. Demographic and Clinical Data of Study Children

The 32 enrolled children, 16 males (50.0%) and 16 females (50.0%) with a median age of 1.4 years (range: 1 day–13 years), underwent NPS at Maternal and Child Health-IRCCS Burlo Garofolo (Trieste, Friuli-Venezia Giulia, Italy) after the onset of symptoms resembling COVID-19. All the subjects tested SARS-CoV-2-positive in NPS (32/32, 100%), with a median viral load of 2.5 × 10^7^ copies/mL (range: 2.5 × 10^3^ –2.5 × 10^8^ copies/mL), as shown in Table 2. The common symptom in almost all included children was fever (31/32, 96.9%). No one was hospitalized, except for a newborn child.

### 3.2. Sequencing of the SARS-CoV-2 RBD Spike Region

SARS-CoV-2 RBD sequences, obtained by means of amplification and subsequent Sanger automatic sequencing, were aligned with the reference SARS-CoV-2 Wuhan-Hu-1 strain (NC_045512.2). The alignment results are reported in Table 3. Briefly, in all sequences (32/32, 100%), the D614G substitution was detected, lineage B.1. A total of 6 out of 32 (18.8%) sequences showed a nucleotide point mutation leading to a synonymous amino acid substitution, while 4/32 (12.5%) sequences carried a total of 5 nucleotide mutations, 3 of which (C22879A) led to the N439K amino acid substitution and 1 of which (G22992A) led to the S477N amino acid substitution. In total, 7/32 (21.8%) strains showed the presence of 3 nucleotide mutations (T22917G, C22995A, C23604G), leading to the L452R, T478K, and P681R amino acid substitutions, all typical of a Delta-like variant. Finally, 1/32 sequence was the Omicron strain, with the following mutations in the RBD domain: T22882G (N440K), G22898A (G446S), G22992A (S477N), C22995A (T478K), A23013C (E484A), A23040G (Q493R), G23048A (G496S), A23055G (Q498R), A23063T (N501Y), T23075C (Y505H), C23202A (T547K), A23403G (D614G), C23525T (H655Y), T23599G (N679K), C23604A (P681H).

The nucleotide mutations and amino acid mutations are shown in Table 4. The nucleotide sequences were deposited in the GISAID EpiCov^TM^ online database.

### 3.3. Full Genome Sequencing of SARS-CoV-2 B.1.258 (∆H69/∆V70) and B.1.617.2+AY.43 by Next-Generation Sequencing

We decided to perform the full genome sequencing of the viral strains that were more represented in the sampled population: the N439K, present in 3/32 patients, and the Delta-like, present in 7/32 patients. Full genome sequences obtained by the NGS of a single SARS-CoV-2 isolated strain, N439K, and a single Delta-like strain, which were deposited at GISAID (N439K: EPI_ISL_7208675; Delta: EPI_ISL_7698448).

The N439K (EPI_ISL_7208675) strain was compared to the reference SARS-CoV-2 genome isolate (Wuhan-Hu-1, NC_045512.2) (Table 4), and it was assigned to lineage B.1.258 (∆H69/∆V70) using Phylogenetic Assignment of Named Global Outbreak Lineages (pangolin) [28] based on the presence of the nonsynonymous mutations or deletions R207H, K1895N, I2501T, M4241I, P4715L, V5112I, H5614Y, and A5922S in the ORF1ab gene; H69del, V70del, G75V, N439K, and D614G in the S gene; and G38stop in the ORF7a gene.

In addition, the Delta-like (EPI_ISL_7698448) strain was compared to the reference SARS-CoV-2 genome isolate (Wuhan-Hu-1, NC_045512.2) (Table 5), and it was assigned to the lineage B.1.617.2 + AY.43 strain using the Pangolin tool [28] based on the presence of the nonsynonymous mutations or deletions A1306S, A1809V, P2046L, P2287S, V2930L, T3255I, T3646A, P4715L, G5063S, L5230I, P5401L, and A6319V in the ORF1ab gene; T19R, G142D, EFR156G (F157Δ, R158Δ), L452R, T478K, D614G, P681R, and D950N in the S gene; S26L in the ORF3a gene; I82T in the M gene; V82A and T120I in the ORF7a gene, T40I in the ORF7b gene; C37R, D119Δ, and F120Δ in the ORF8 gene; and Q9L, D63G, R203M, G215C, and D377Y in the N gene.

### 3.4. Phylogenetic Analysis

The phylogenetic analysis of 31/32 study SARS-CoV-2 nucleotide sequences revealed an intragroup nucleotide identity of 99.6% (range: 97.5–100%) and a mean genetic distance (*p*-distance) of 0.004 ± 0.010. This analysis demonstrates that the study sequences were genetically related.

**Figure 1:**
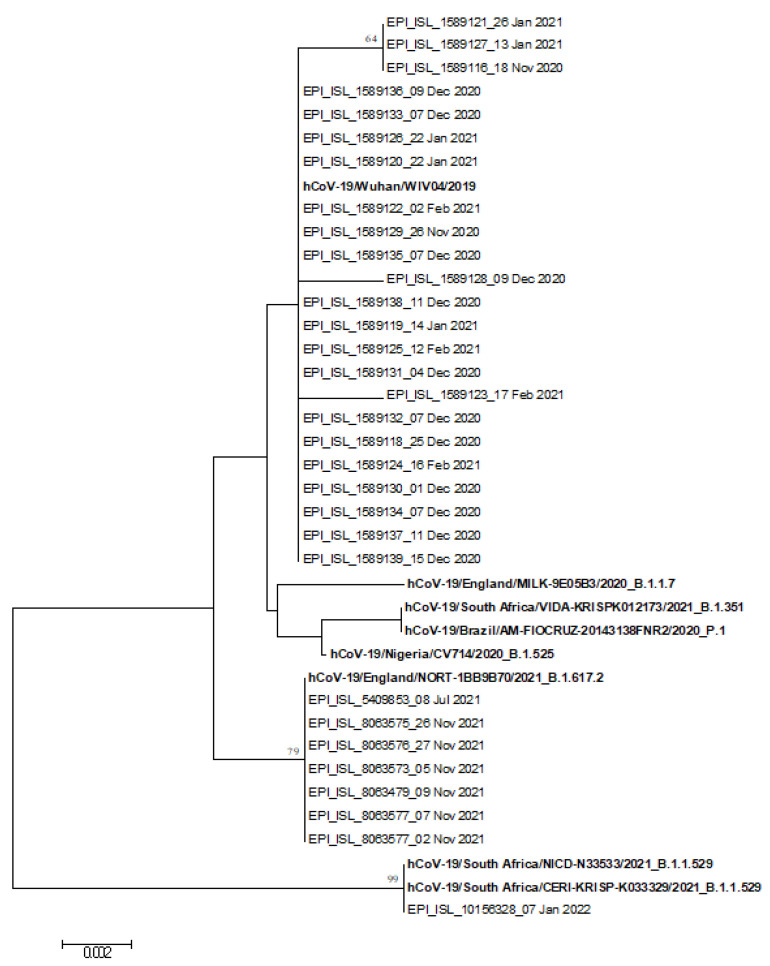
Phylogenetic tree of the 31 S gene nucleotide sequences of study SARS-CoV-2 strains identified in Trieste and reference strains (in bold).

However, as shown in the phylogenetic tree in Figure 1, the study sequences were distributed in three major clades. In total, 23/31 sequences were “no-VOC,” showing an intragroup nucleotide identity of 99.9% (range: 99.5–100%) and a *p*-distance of 0.001 ± 0.000. These sequences were strictly related each other and were detected from November 2020 to February 2021. In addition, 7/31 sequences belonged to the lineage B.1.617.2, also known as Delta VOC. These sequences showed an intragroup nucleotide identity of 100%, and were detected in July 2021 (1/7) and in November 2021 (6/7). Finally, 1/31 sequences belonged to the lineage B.1.1.529, also known as Omicron VOC. This sequence was detected in January 2022.

### 3.5. SARS-CoV-2 Isolates Growth Curves

**Figure 2:**
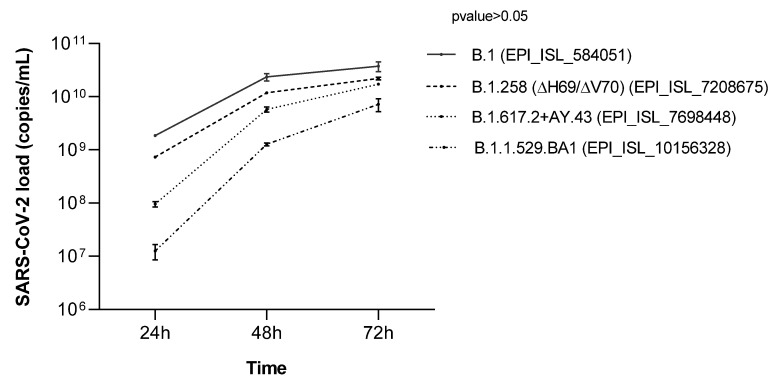
Growth curves of isolated variants, B.1.258 (∆H69/∆V70) (GISAID ID: EPI_ISL_7208675) and B.1.617.2+AY.43 (GISAID ID: EPI_ISL_7698448), and B.1.1.529.BA1 (EPI_ISL_10156328) compared with B.1, previously isolated (GISAID ID: EPI_ISL_584051). The viral loads, expressed as copies/mL, were quantified in supernatants by means of RT–qPCR targeting the SARS-CoV-2 N gene at 24, 48, and 72 h post-infection. The growth curves showed the same trend, with an increase in viral load from 24 to 72 h.

Isolation and growth curves of SARS-CoV-2 strains carrying nonsynonymous amino acid mutations were performed. A comparison was performed with the SARS-CoV-2 B.1 strain (EPI_ISL_584051) previously isolated in our laboratory [29]. No significant differences were observed between the four growth curves (*p* value > 0.05) (Figure 2). The viral loads increased starting from 24 h post-infection for the four strains.

### 3.6. Microneutralization Assay

**Figure 3:**
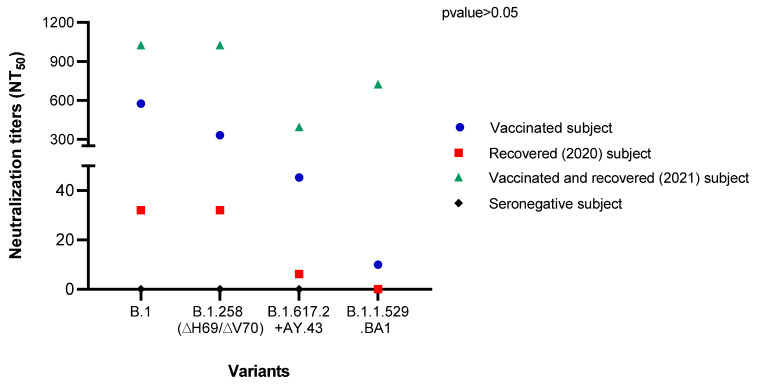
Serum neutralizing titers NT_50_ of a COVID-19 vaccinated subject, recovered (2020) patient, vaccinated and recovered (2021) patient, and seronegative subject, against B.1 (GSAID ID: EPI_ISL_584051), B.1.258 (∆H69/∆V70) (GISAID ID: EPI_ISL_7208675), B.1.617.2+AY.43 strain (GISAID ID: EPI_ISL_7698448), and B.1.1.529.BA1 (GISAID ID: EPI_ISL_10156328). NT_50_ values of sera were obtained by microneutralization assay and determined by means of the Reed and Muench method.

The results obtained from the microneutralization assay were evaluated by inspecting the inhibition of the CPE for each serum dilution by an inverted optical microscope. The NT_50_ for the SARS-CoV-2 tested strains, mixed with the sera from a Comirnaty (Pfizer-BioNTech) COVID-19 vaccinated subject, COVID-19 patient recovered in 2020, patient who was infected after the third dose of Comirnaty (Pfizer-BioNTech) and recovered recently (December 2021), and seronegative patient were determined by means of the Reed and Muench method (Figure 3). NT_50_ values against the B.1.258 (∆H69/∆V70) (GISAID ID: EPI_ISL_7208675), B.1.617.2+AY.43 (GISAID ID: EPI_ISL_7698448), and B.1.1.529.BA1 (EPI_ISL_10156328) strain were 1.7-, 12.7-, and 57.6-fold lower than against B.1 in a COVID-19 vaccinated subject, respectively. In the recovered COVID-19 patient in 2020, the NT_50_ value was the same in B.1 (GISAID ID: EPI_ISL_584051) and B.1.258 (∆H69/∆V70) (GISAID: EPI_ISL_7208675), while it was 5.2-fold lower in B.1.617.2+AY.43 (GISAID: EPI_ISL_7698448), and no neutralization was observed against B.1.1.529.BA1 (EPI_ISL_10156328). In the recently recovered patient, NT_50_ values against B.1 (GISAID ID: EPI_ISL_584051) and B.1.258 (∆H69/∆V70) (GISAID: EPI_ISL_7208675) were higher than 1024. A reduction of 2.5- and 1.4-fold of the NT_50_ value against B.1.617.2+AY.43 (GISAID: EPI_ISL_7698448) and B.1.1.529.BA1 (EPI_ISL_10156328), respectively, was observed compared to the B.1 strain. In the seronegative patient, no neutralization against the employed variants was observed.

In summary, a reduction in serum neutralizing activity against variants was observed, especially against B.1.617.2+AY.43 (GISAID ID: EPI_ISL_7698448) and B.1.1.529.BA1 (EPI_ISL_10156328), but the differences were not significant (*p* value > 0.05).

## 4. Discussion

The continuing spread of SARS-CoV-2 in the human population induces the emergence of new variants that could impact the epidemiological situation of geographical areas where these variants become predominant. The transmissibility, severity of these strains, and immune response to the infection may be altered [30]. Children are a poorly studied population, and very few studies have investigated the circulation of SARS-CoV-2 and its variants in this population. Children are reservoirs of the virus, contributing to the spread of SARS-CoV-2 variants [16]. Currently, COVID-19 vaccines are available and recommended for children aged 5 and above [31], but no vaccines have been authorized for children aged 0–4 years.

In this regard, the focus of this paper was to analyze the sequences of SARS-CoV-2 isolated from SARS-CoV-2-positive NPSs collected from 32 children to investigate the circulation of variants in a geographic area of northeastern Italy.

The patients were all infected with the SARS-CoV-2 B.1 variant, which appeared in North Italy in April 2020 and was the predominant variant, together with its derivatives, until February 2021 [32]. The B.1 strain shows the D614G substitution, able to confer moderate advantages in viral infectiveness and transmissibility, compared to the Wuhan-Hu-1 strain [33,34].

Among the isolates, the N439K substitution was found in three samples. Based on the Pangolin nomenclature, the SARS-CoV-2 strain presenting this mutation was assigned to the B.1.258 lineage. The strain was detected for the first time in Scotland in March 2020 [35], and spread quickly in many European countries [36]. The N439K substitution occurs in the RBD, the highly variable region of the SARS-CoV-2 spike protein, which interacts with ACE2 receptors for viral entry and is the main target of neutralizing antibodies [36,37,38]. This mutation seems to increase the affinity of virus S protein to the ACE2 receptor, and reduce the activity of designed monoclonal antibodies and serum polyclonal antibodies from recovered COVID-19 patients [35,36]. Consequently, N439K might evade the immune system either in recovered COVID-19 patients or in vaccinated subjects. Our results are in line with the published literature, indicating that the neutralizing activity of sera from the COVID-19 vaccinated subject decreased against the B.1.258 strain up to two-fold compared to the B.1 strain.

NGS also revealed the presence of the Δ69/70 mutation in the B.1.258 strain, a mutation that emerged independently in the other five strains (namely B.1.1.7, B.1.1.298, B.1.160, B.1.177, B.1.375), always after the acquisition of receptor binding motif replacements, such as N439, in several European countries in the beginning of August 2020. During the fall of 2020, the B.1.258 variant gained significant prevalence in multiple countries, and Brejova and colleagues speculated that its diffusion was associated with increasing hospitalization trends, starting before the appearance of the B.1.1.7 variant [39]. Different in vitro studies have reported contrasting results regarding the increased replication of the strains carrying the deletion [40,41]. In this regard, we did not observe a significant difference in the replication trend of the isolated SARS-CoV-2 strains.

Of note, NGS analysis also revealed the presence of a stop codon in the ORF7a coding region (G58) due to the G/T mutation at nt 27505. ORF7a, together with ORF3, ORF6, and ORF8, is an accessory viral protein involved in SARS-CoV-2 immune escape mechanisms since they are able to antagonize type I IFN production and signaling [42,43,44].

Mutations are not infrequent in the ORF7 region. Nemudriy and colleagues reported that C-terminal mutations are often reported in patients worldwide. In particular, mutations leading to the truncation of the ORF7a products have been previously observed, and they have led to a loss of protein function that abrogates its interaction with IFN signaling [45].

To the best of our knowledge, this is the first study to report the detection of the N439K substitution in a pediatric population. No significant differences were observed among the clinical outcomes of the three children positive for N439K and the others, confirming the findings reported by Thomson and colleagues, who found no association between this substitution and increases in COVID-19 symptomatology and severity [36]. In contrast, we did not find any differences in the viral load between the N439K and D614G strains [36].

The variant carrying the S477N substitution spread in some European countries, with a higher prevalence in France during the summer of 2020 [46] and a frequency of detection ranging from 4–7% [47]. In our study, the S477N substitution was detected in one sample, with a frequency of 3.1%, resembling its frequency rate in Europe, which ranges from 4 to 7% [47]. As for N439K, S477N occurs in the RBD region and seems to confer enhanced binding ability to the ACE2 receptor [35], but it does not seem to be associated with the loss of neutralizing activities of convalescent serum antibodies [35,48,49].

In our cohort, and compared to the D614G strain, the S477N viral load was slightly lower, while no difference in the clinical presentation was observed.

Seven isolates in our cohort of children were Delta isolates collected from July 2021 to November 2021. Starting in May 2021, the Delta variant became the predominant variant in many European countries, drastically enhancing the spread of the virus and causing a high number of infections [50]. The clinical symptoms of SARS-CoV-2 Delta-positive children were similar to those affecting other positive children, suggesting that the variant does not affect the clinical course in the pediatric population independently by the variant [51].

As reported in another study [52], we observed a marked reduction (up to almost 13-fold) in the effectiveness of the Comirnaty vaccine against the delta-like variant in vitro. A decrease in the neutralization ability against this variant was also observed testing the sera from both the recovered and the vaccinated and recovered patients (up to 5-fold and up to 2.5-fold, respectively).

Since January 2022, the Omicron variant has become the predominant one in Italy, as well as the rest of Europe. We collected the NPS from one asymptomatic, Omicron-infected child, and we observed that the B.1.1.529.BA1 variant could be neutralized only after the booster dose of vaccine, as already reported in the literature [53,54]. No or very weak neutralization was indeed observed in the recovered patient, and in the subject with two doses of vaccine, respectively.

## 5. Conclusions

Although we are aware that the conclusions arose from a limited number of patients, it should be underlined that studying the pediatric population is not simple, since children have a smaller number, lower incidence, and milder symptoms than adult patients [55,56].

Further investigations on a large group of children are needed to assess the circulation of SARS-CoV-2, especially because children contribute to the spread of the virus like adults. The vaccination of young people will be an important step to stop the spread of the virus.

## Figures and Tables

**Table 1:** RBD primers positions and sequences.

Primer	Position *	Sequences (5′-3′)
**SARS-CoV-2 S-F5**	22775-22801	GATGAAGTCAGACAAATCGCTCCAGG
**SARS-CoV-2 S-R6**	23641-23668	TGCCTACACTATGTCACTTGGTGCAGAA

* Nucleotide positions refer to the SARS-CoV-2 isolate Wuhan-Hu-1 complete genome (NC_045512.2).

**Table 2:** Demographic and clinical data of SARS-CoV-2-positive study children.

Patient	Gender	Age	Clinical Symptoms	Viral Load (Copies/mL)
#1	F	13 years	Fever	2.50 × 10^7^
#2	F	11 years	Fever	2.50 × 10^6^
#3	M	8 months	Neutropenic fever	2.50 × 10^8^
#4	M	15 months	Fever	2.50 × 10^7^
#5	F	14 months	Fever	2.50 × 10^7^
#6	F	1 month	Fever	2.50 × 10^7^
#7	F	21 months	Fever	2.50 × 10^6^
#8	F	7 months	Fever	2.50 × 10^7^
#9	M	8 months	Fever, rhinitis	2.50 × 10^5^
#10	F	5 months	Fever	2.50 × 10^8^
#11	M	20 months	Fever	2.50× 10^5^
#12	F	13 months	Fever	2.50 × 10^5^
#13	M	11 months	Fever	2.50 × 10^4^
#14	M	4 months	Fever	2.50 × 10^5^
#15	F	8 years	Fever, pharyngodynia	2.50 × 10^8^
#16	M	2 months	Fever	2.50 × 10^7^
#17	M	13 years	Fever	2.50 × 10^8^
#18	M	10 years	Fever	2.50 × 10^7^
#19	F	10 years	Fever	2.50 × 10^7^
#20	F	13 years	Fever, headache	2.50 × 10^7^
#21	F	11 years	Fever	2.50 × 10^8^
#22	F	10 years	Fever, headache	2.50 × 10^8^
#23	M	13 years	Fever, vomit	2.50 × 10^7^
#24	F	13 years	Fever	2.50 × 10^8^
#25	M	7 years	Fever	2.50 × 10^8^
#26	M	1 day	Fever	2.50 × 10^3^
#27	F	3 months	Fever	2.50 × 10^7^
#28	M	11 years	Fever	2.50 × 10^7^
#29	M	18 months	Fever	2.50 × 10^7^
#30	M	2 months	Fever	2.50 × 10^7^
#31	F	9 years	Fever	2.50 × 10^7^
#32	M	12 years	Asymptomatic	2.50 × 10^7^

**Table 3:** SARS-CoV-2 RBD sequence analysis of enrolled children, with GISAID ID.

Sequence ID	Nucleotide Mutations	Amino Acid Mutations	GISAID ID
#1	C22987T, A23403G	Synon, D614G	EPI_ISL_1589116
#2	G22992A, A23403G	S477N, D614G	EPI_ISL_1589117
#3	A23403G	D614G	EPI_ISL_1589118
#4	A23403G	D614G	EPI_ISL_1589119
#5	A23403G	D614G	EPI_ISL_1589120
#6	C22987T, Ins C (23056) A23403G	Synon, - D614G	EPI_ISL_1589121
#7	A23403G	D614G	EPI_ISL_1589122
#8	T23047C, A23403G	Synon, D614G	EPI_ISL_1589123
#9	C22879A, A23403G	N439K, D614G	EPI_ISL_1589124
#10	C22879A, A23403G	N439K, D614G	EPI_ISL_1589125
#11	A23403G	D614G	EPI_ISL_1589126
#12	C22987T, A23403G	Synon, D614G	EPI_ISL_1589127
#13	C23230T, A23403G	Synon, D614G	EPI_ISL_1589128
#14	A23403G	D614G	EPI_ISL_1589129
#15	A23403G	D614G	EPI_ISL_1589130
#16	A23403G	D614G	EPI_ISL_1589131
#17	A23403G	D614G	EPI_ISL_1589133
#18	A23403G	D614G	EPI_ISL_1589135
#19	T22831C, A23403G	Synon, D614G	EPI_ISL_1589134
#20	A23403G	D614G	EPI_ISL_1589132
#21	A23403G	D614G	EPI_ISL_1589136
#22	A23403G	D614G	EPI_ISL_1589138
#23	A23403G	D614G	EPI_ISL_1589137
#24	C22747T, C22879A A23403G	Synon, N439K D614G	EPI_ISL_1589139
#25	T22917G, C22995A A23403G, C23604G	L452R, T478K D614G, P681R	EPI_ISL_5409853
#26	T22917G, C22995A A23403G, C23604G	L452R, T478K D614G, P681R	EPI_ISL_8063575
#27	T22917G, C22995A A23403G, C23604G	L452R, T478K D614G, P681R	EPI_ISL_8063576
#28	T22917G, C22995A A23403G, C23577T, C23604G	L452R, T478K D614G, A672V P681R	EPI_ISL_8063573
#29	T22917G, C22995A A23403G, C23604G	L452R, T478K D614G, P681R	EPI_ISL_8063479
#30	T22917G, C22995A A23403G, C23604G	L452R, T478K D614G, P681R	EPI_ISL_8063577
#31	T22917G, C22995A A23403G, C23604G	L452R, T478K D614G, P681R	EPI_ISL_8063577
#32	T22882G, G22898A G22992A, C22995A A23013C, A23040G G23048A, A23055G A23063T, T23075C C23202A, A23403G C23525T, T23599G C23604A	N440K, G446S S477N, T478K E484A, Q493R G496S, Q498R N501Y, Y505H T547K, D614G H655Y, N679K P681H	EPI_ISL_10156328

Synon: synonym mutation; Ins: insertion.

**Table 4:** Nucleotide mutations and amino acid substitutions in B.1.258 (∆H69/∆V70) (EPI_ISL_7208675) compared to the Wuhan strain (NC_045512).

Nucleotide Position	EPI_ISL_7208675	Reference NC_045512	Gene	Variant Type	Amino Acid Change
241	C	T	Intergenic		Non coding
885	G	A	ORF1ab	Non syn	R207H
3037	C	T	ORF1ab	Syn	F924
5950	G	T	ORF1ab	Non syn	K1895N
7767	T	C	ORF1ab	Non syn	I2501T
8047	C	T	ORF1ab	Syn	Y2594
12,988	G	T	ORF1ab	Non syn	M4241I
14,064	T	C	ORF1ab	Syn	D4600
14,408	C	T	ORF1ab	Non syn	P4715L
15,598	G	A	ORF1ab	Non syn	V5112I
17,104	C	T	ORF1ab	Non syn	H5614Y
18,028	G	T	ORF1ab	Non syn	A5922S
19,032	C	T	ORF1ab	Syn	D6256
20,268	A	G	ORF1ab	Syn	L6668
21,764	ATACATG	A	S	Deletion	H69Δ, V70Δ
21,786	G	T	S	Non syn	G75V
22,747	C	T	S	Syn	V395
22,879	C	A	S	Non syn	N439K
23,403	A	G	S	Non syn	D614G
24,910	T	C	S	Syn	T1116
26,972	T	C	M	Syn	R150
27,505	G	T	ORF7a	Stop	G38 *
27,800	C	A	ORF7b	Syn	A15
29,734	G	C	Intergenic		Non coding

Syn: synonymous mutation; Non syn: nonsynonymous mutation; Δ: deletion; *: stop codon.

**Table 5:** Nucleotide mutations and amino acid substitutions in the B.1.617.2+AY.43 strain (GISAID ID: EPI_ISL_7698448) compared to the Wuhan strain (NC_045512).

Nucleotide Position	EPI_ISL_7698448	Reference NC_045512	Gene	Variant Type	Amino Acid Change
210	T	G	Intergenic		Non coding
241	T	C	Intergenic		Non coding
3037	T	C	ORF1ab	Syn	F924
4181	T	G	ORF1ab	Non syn	A1306S
5691	T	C	ORF1ab	Non syn	A1809V
6402	T	C	ORF1ab	Non syn	P2046L
7124	T	C	ORF1ab	Non syn	P2287S
8986	T	C	ORF1ab	Syn	D2907
9053	T	G	ORF1ab	Non syn	V2930L
10,029	T	C	ORF1ab	Non syn	T3255I
11,201	G	A	ORF1ab	Non syn	T3646A
11,332	G	A	ORF1ab	Syn	V3689
14,408	T	C	ORF1ab	Non syn	P4715L
15,451	A	G	ORF1ab	Non syn	G5063S
15,952	A	C	ORF1ab	Non syn	L5230I
16,466	T	C	ORF1ab	Non syn	P5401L
19,220	T	C	ORF1ab	Non syn	A6319V
21,618	G	C	S	Non syn	T19R
21,987	A	G	S	Non syn	G142D
22,028	G	GAGTTCA	S	Deletion	EFR156G F157Δ, R158Δ
22,917	G	T	S	Non syn	L452R
22,995	A	C	S	Non syn	T478K
23,403	G	A	S	Non syn	D614G
23,604	G	C	S	Non syn	P681R
24,124	A	G	S	Syn	K854
24,410	A	G	S	Non syn	D950N
25,469	T	C	ORF3a	Non syn	S26L
26,767	C	T	M	Non syn	I82T
27,638	C	T	ORF7a	Non syn	V82A
27,752	T	C	ORF7a	Non syn	T120I
27,874	T	C	ORF7b	Non syn	T40I
28,002	C	T	ORF8	Non syn	C37R
28,247	A	AGATTTC	ORF8	Deletion	D119Δ, F120Δ
28,270	T	TA	Intergenic		Non coding
28,299	T	A	N	Non syn	Q9L
28,461	G	A	N	Non syn	D63G
28,881	T	G	N	Non syn	R203M
28,916	T	G	N	Non syn	G215C
29,402	T	G	N	Non syn	D377Y
29,742	T	G	Intergenic		Non coding

Syn: synonymous mutation; Non syn: nonsynonymous mutation; Δ: deletion.

## Data Availability

All datasets generated for this study are included in the article. Genome sequence with detailed information of the strains is available in the GISAID database, with the accession numbers reported in the text.
